# Editorial: Rising stars in microbiotechnology: 2022

**DOI:** 10.3389/fmicb.2023.1347005

**Published:** 2024-01-04

**Authors:** Saurabh Sudha Dhiman, Kian Mau Goh

**Affiliations:** ^1^Chemistry, Biology and Health Sciences, South Dakota Mines, Rapid City, SD, United States; ^2^Civil and Environmental Engineering, South Dakota Mines, Rapid City, SD, United States; ^3^Department of Biosciences, Faculty of Science, Universiti Teknologi Malaysia, Skudai, Johor, Malaysia

**Keywords:** *Acidithiobacillus ferrooxidans*, biodegradation, extracellular polymeric substances, *Methylococcus capsulatus*, microalgal-bacterial consortia, polycyclic aromatic hydrocarbons, wine fermentation

Microbiotechnology has the potential to revolutionize an array of areas, including but not limited to medicine, agriculture, energy, and environmental preservation. Within this Research Topic, we present the remarkable contributions of emerging researchers in the field of microbiotechnology. Their work sheds light on theoretical, experimental, and methodological advancements, all of which have practical applications addressing significant challenges.

For decades, DNA extraction protocols have been developed and commercialized. However, these kits often come at a considerable cost, especially when processing a large number of samples for PCR amplification, where modest concentration and purity suffice. In a recent report, featured in the Methods section, the authors introduced a straightforward, expeditious, and cost-effective methodology for extracting DNA from pure cultures. This innovative technique entails subjecting the samples to 0.1 M potassium hydroxide at a temperature of 100°C for a duration of 10 min, followed by centrifugation, as detailed by Liu et al. The resulting templates have been shown to work well for a variety of microorganisms, and the amplification efficiencies are comparable to or better than those obtained using other methods.

*Methylococcus capsulatus* Bath, a methane-oxidizing bacterium, harbors vast potential for applications in bioremediation, biotechnology, and bioenergy production. However, its performance in these applications is substantially influenced by abiotic variables. In an endeavor to gain a deeper understanding of the adaptability of this bacterium to varying surroundings, researchers have investigated its metabolic responses using RNA sequencing and GC-MS (Bedekar et al.). The authors identified crucial genes and pathways involved in energy production, nitrogen metabolism, and carbon fixation. For example, the authors found that *M. capsulatus* upregulated the expression of genes involved in methane oxidation and carbon fixation at low temperatures. Furthermore, the research unveiled that *M. capsulatus* favors assimilation of nitrate as its preferred nitrogen source, but it can transition to ammonium assimilation when faced with nitrogen scarcity.

*Acidithiobacillus ferrooxidans*, renowned for its ability to oxidize ferrous iron and sulfur compounds, plays a pivotal role in biomining operations, extracting valuable metals from ores. The attachment of *A. ferrooxidans* to the ore surface is essential for biomining. One of the studies in this Research Topic investigated the effect of galactose and high ferric iron concentration on the production of extracellular polymeric substances (EPS) and the attachment of the cells on a polymetallic sulfide ore surface (Moncayo et al.). The authors found that the combined effect of galactose and ferric iron concentration significantly increased the formation of EPS and enhanced cell adhesion to the ore surfaces. We anticipate that this research will set the stage for future studies on the sustainability of increased bacterial attachment and its potential to extend biooxidation and bioleaching applications.

Polycyclic aromatic hydrocarbons (PAHs), a class of environmental pollutants posing substantial risks to both human health and ecosystems, are candidates for bioremediation. Hoque et al. investigated the biodegradation of phenanthrene and anthracene, two common PAHs, using a consortium comprising the microalga *Gonium pectorale* and the bacterium *Bacillus licheniformis* (Hoque et al.). The consortium demonstrated superior efficacy in degrading both PAHs compared to either species alone, suggesting a synergistic effect. The authors proposed a possible mechanism for the enhanced biodegradation, involving the microalgae providing organic carbon and other nutrients for the bacteria, while the bacteria provide essential nutrients for the microalgae. This study offers invaluable insights into the potential of microalgal-bacterial consortia for the bioremediation of PAHs.

Wine alcoholic fermentation is a complex process that requires careful optimization to produce top-tier wines. Sterols, a class of lipids, play a vital role in yeast cell membrane integrity and function during fermentation. Girardi-Piva et al. examined the effects of two types of sterols, ergosterol and phytosterol, on wine fermentation, employing an extensive array of wine yeast strains and manipulating sugar concentrations. Their findings suggest that sterol assimilation, especially phytosterols, can enhance yeast growth, metabolism, and viability during fermentation, leading to improved fermentation efficiency and a reduced risk of sluggish or stuck fermentations. The authors further posited the potential active pathways under sterol scarcity and high sugar content conditions, albeit affirming that these speculations require further research. Overall, the study by Girardi-Piva et al. underscores the pivotal role of sterols in wine fermentation and provides fresh insights into harnessing sterols to enhance fermentation performance.

Perchloroethylene (PCE) is a highly toxic, carcinogenic, and mutagenic contaminant. PCE has the potential to persistently contaminate groundwater. Harnessing the power of synthetic microbiomes and controlled microbial biofilms within bioelectrochemical systems (BESs) could revolutionize the remediation of PCE-contaminated sites. BESs utilize microorganisms to degrade PCE under the influence of an electric field. In this Research Topic, Di Franca et al. investigated the microbiome composition and dynamics within a novel BES designed for PCE removal. The BES was able to remove PCE from all feeding compositions; the removal rate was fastest in the BES fed with anaerobic mineral medium (MM), followed by the BES fed with synthetic groundwater (SG) and the BES fed with real groundwater (RG). Moreover, the authors found that the presence of sulfate and nitrate in SG and RG inhibited the growth of key bacteria involved in PCE removal, particularly *Sulfuricurvum* and *Mycobacterium*. The bacterium *Sulfuricurvum*, a sulfur-oxidizing microbe, plays a crucial role in the de-chlorination process, while *Mycobacterium* is responsible for the aerobic degradation of vinyl chloride, a byproduct of PCE degradation. This research sets the stage for future investigations, including efforts to manipulate BES microbiome composition to optimize PCE removal and the evaluation of BES performance in pilot-scale and field-scale scenarios.

This Research Topic serves as a helpful resource for readers with an interest in staying abreast of the most recent advancements in the field of microbiotechnology. This exhibition presents compelling research conducted by up-and-coming scholars in the discipline, emphasizing the transformative capacity of microbes that have a tangible impact on the scientific community.

## Author contributions

SD: Writing—review & editing. KG: Writing—review & editing, Writing—original draft.

